# Partograph Utilization and Associated Factors among Obstetric Care Providers Working in Public Health Facilities of Wolaita Zone, 2017

**DOI:** 10.1155/2020/3631808

**Published:** 2020-07-01

**Authors:** Mesfin Markos, Aseb Arba, Kebreab Paulos

**Affiliations:** ^1^Department of Midwifery, Wolaita Sodo University College of Medicine and Health Sciences, Ethiopia; ^2^Department of Nursing, Wolaita Sodo University College of Medicine and Health Sciences, Ethiopia

## Abstract

**Background:**

Obstructed or prolonged labor is a major cause of maternal deaths. Prolonged and obstructed labor contributed to 13% of global maternal deaths which can be reduced by proper utilization of a partograph during labor. Obstetric caregivers' use of the partograph during labor has paramount importance in identifying any deviation during labor. Even though partograph use is influenced by different factors as obtained from the literatures, the magnitude of partograph utilization and the factors associated with its use are not well determined in the health facilities of Wolaita Zone.

**Objective:**

To assess the magnitude of partograph utilization and factors that affect its utilization among obstetric caregivers in public health facilities of Wolaita Zone, Ethiopia, 2017.

**Methods:**

An institution-based cross-sectional study was conducted on obstetric caregivers. A pretested and structured questionnaire was used to collect data. Data was entered to EpiData version 3.01 and exported to SPSS version 23.0 for further analysis. Logistic regression analyses were used to see the association of different variables.

**Result:**

A total of 269 obstetric caregivers participated in the study. Among those who were utilizing the partograph, 193 (71.7%) routinely used it for all laboring mothers and 76 (28.3%) of participants reported that they do not routinely utilize it. Greater number of service years (AOR = 4.93, 95% CI: 1.53-15.88), on-the-job training (AOR = 0.16, 95% CI: 0.06-0.43), good knowledge (AOR = 3.35, 95% CI: 1.61-6.97), and favorable attitude towards partograph utilization (AOR = 2.99, 95% CI: 1.28-7.03) were significantly associated with partograph utilization. *Conclusion and Recommendation*. Partograph utilization among obstetric caregivers in the public health facilities was good. Greater years of work experience, in-service training, having good knowledge, and favorable attitude towards partograph utilization among obstetric caregivers independently determined partograph utilization. Provision of on-the-job training to make obstetric caregivers improve knowledge and skill on partograph utilization, maintaining caregivers' retention to decrease turnover by providing different incentives to more experienced obstetric care providers, and establishing favorable attitude could improve the proper use of the tool.

## 1. Introduction

The partograph is the graphic recording of the progress of labor and the salient condition of the mother and the fetus. It serves as an “early warning system” and assists in early decision to action. A partograph is universally used as part of the Safe Motherhood Initiative for improving labor management and reducing maternal and fetal morbidity and mortality [1]. Around the world, there were an estimated 303,000 maternal deaths in 2015, in which every day, approximately 830 women die from avoidable causes related to pregnancy and childbirth. The overall MMR in developing regions is 239 maternal deaths per 100,000 live births, which is roughly 20 times higher than that of developed regions. About 99% of maternal deaths occur in developing countries, while more than half of these deaths occur in sub-Saharan Africa, in which most of them were preventable. The global lifetime risk of maternal mortality is approximately 1 in 180. Maternal death is the major public health problem of developing countries [2].

Although different governmental and nongovernmental organizations play their role to decrease maternal death in Ethiopia, still, the expected goal was not met. The Ethiopian Demographic and Health Survey (EDHS) 2016 shows that the ratio of maternal mortality in Ethiopia is 412/100,000 live births [3]. The considerable causes of maternal deaths in Ethiopia are similar to most developing countries: obstructed labor, infection, hemorrhage, abortion, and hypertension in pregnancy. The proportion ascribed to the different causes varies from year to year. The majority of maternal deaths and complications were attributable to obstructed and prolonged labor [4].

Using partograph for early detection and timely intervention of obstetric complications is the most important activity to prevent maternal and perinatal mortality and morbidity [5]. Interventions that can prevent complications from the major causes of preventable death are known and can be made available even in resource-poor settings. The partograph is one of the strongest and cost-effective tools to prevent unnecessary delay and serves as a basic tool for obstetric caregivers [6].

The World Health Organization (WHO) recommends using the partograph to follow labor and delivery, with the objective to improve health care and reduce maternal and fetal morbidity and death [2, 5, 7]. In spite of government commitment for the reduction of maternal death by more than three quarters over the period of 1990 to 2016, maternal mortality ratio remains high [3].

Obstructed or prolonged labor significantly accounted for maternal deaths, and women with obstructed labor usually suffer from postpartum hemorrhage, uterine rupture, puerperal sepsis, and obstetric fistula in Ethiopia. Based on a study conducted in Jimma University Specialized Hospital, obstructed labor, cephalopelvic disproportion, and malpresentation were the main causes of prolonged labor which in turn leads to uterine rupture (45.1%), the commonest complication of obstructed labor [8–11].

Based on finding of a study conducted in Mizan-Aman Hospital, the prevalence of maternal mortality during childbirth was 13(3.39%). The causes of death were obstructed labor, puerperal sepsis, multipregnancy, hypertensive disorder, hemorrhage, and IUFD. These complications could have been easily detected and managed if correctly followed with a partograph [9].

Proper use of the partograph during delivery is for achieving a healthy mother and baby with the least possible level of intervention, early detection, and management of complications and timely referral. And the partograph is a vital available tool for caregivers who need to be able to identify complications in childbirth in a timely manner and refer women to an appropriate facility for treatment. Thus, prevention of prolonged and obstructed labor by using the partograph during labor is a key intervention in the reduction of maternal and perinatal morbidity and mortality [10, 12, 13].

Studies conducted in African countries have shown that the prevalence of partograph use is low despite preparing a tool that is simple and inexpensive for intrapartum monitoring of labor [14]. Similarly, studies conducted in North Shoa, Ethiopia, revealed low utilization of the partograph [15]. The lack of the preprinted partograph in the health facilities, profession type, getting on-the-job training, and poor knowledge and attitude towards the partograph were reasons for not using the partograph during labor. However, it was tried to resolve all these challenges to the use of the partograph by provisions of preservice and in-service training on the partograph [15–17].

Little is known about the magnitude of partograph utilization and its associated factors in the study area; understanding this will help policy makers, stakeholders, program planners, and obstetric caregivers to improve the quality of intrapartum care.

### 1.1. Significance of the Study

Three delays are common in Ethiopia; these are delays in recognizing problems and deciding to seek care, delays in transportation to reach appropriate care facilities, and delays in receiving appropriate care at the health facility. Partograph use plays an indispensable role to identify problems, recognize complications early, and perform basic interventions [7].

So, results of the study help to inform areas where supportive supervision is needed. Ultimately, the stakeholders will get adequate information on the magnitude of partograph utilization and act on gap areas.

### 1.2. Objectives

- To determine the magnitude of partograph utilization by obstetric staff in maternity units of public health facilities of Wolaita Zone, Ethiopia, 2017
- To identify factors associated with partograph utilization among obstetric caregivers in public health facilities of Wolaita Zone, Ethiopia, 2017

## 2. Methods and Materials

### 2.1. Study Area and Study Period

The study was conducted from February to April 2017 in Wolaita Zone. The capital city of the zone is about 327 km away from Addis Ababa. In Wolaita Zone are currently 5 governmental and 2 nongovernmental hospitals, 342 health posts, 10 private clinics, and 67 health centers aged 5 years or above as of establishment. In Wolaita Zone, the total number health caregivers are 2787; among these, 770 caregivers give delivery service and 878 are health extension workers as per the Wolaita Zone health department record.

### 2.2. Study Design

A health facility-based quantitative cross-sectional study design was conducted.

### 2.3. Population

The source populations were all obstetric caregivers in public health facilities at Wolaita Zone. All obstetric caregivers who are working in the maternity unit of selected health facilities were considered as the study population.

### 2.4. Eligibility Criteria

All obstetric caregivers who were working as maternity caregivers in the sampled health facilities were included in study. Obstetric caregivers on sick leave and annual leave and students were excluded from the study.

### 2.5. Sample Size Determination

Sample size was determined by using a single population proportion formula taken from the magnitude of partograph utilization study in North Shewa, Northern Ethiopia; the magnitude of partograph utilization among health caregivers was 40.2%, using 95% confidence interval and margin of error 0.05. The initial sample size was 369.4, and a correction formula was used because the population size was less than 10,000. The sample size after the correction formula was 250, and after adding a 10% nonresponse rate, the total sample size for this study was 275.

### 2.6. Sampling Procedure

First, the selection of public health facilities was done by using a simple random sampling method after stratifying them to the level of service they give. There were seven hospitals and 67 health centers. From them, 4 hospitals and 12 health centers were selected randomly to meet the sample size. All obstetric caregivers in the selected public health facility were considered as study participants.

### 2.7. Study Variables

#### 2.7.1. Dependent Variable

- Partograph utilization

#### 2.7.2. Independent Variable

- *Sociodemographic characteristics*: sex, age, service year, and level of education
- *Obstetric caregivers' factors*: knowledge, attitude, and training
- *Health facility factors*: work load, supervision, availability of partograph, and place of work

### 2.8. Operational Definition

*Partograph utilization*: obstetric caregivers who have been using partograph routinely for all laboring mother.

*Obstetric caregivers*: the caregivers include a medical doctors, midwiferies, nurses, and health officers who give labor follow up and delivery service.

*Not utilizing partograph*: obstetric caregivers who were not routinely using a partograph.

*Good knowledge*: obstetric caregivers who score 50% and above of 6 knowledge questions in the questionnaire.

*Poor knowledge*: obstetric caregivers who score less than 50% of 6 knowledge questions in the questionnaire.

*Favorable attitudes*: obstetric caregivers who score a mean value and above of seven attitude questions in the questionnaire.

*Unfavorable attitudes*: obstetric caregivers who score less than the mean value of seven attitude questions in the questionnaire.

### 2.9. Data Collection Tools and Procedure

A pretested and structured self-administered questionnaire was used for data collection. The questionnaire was adapted by reviewing different literatures [11, 15, 17–24]. Data were collected by four experienced midwives from a nonselected institution and one midwife with master's degree qualification supervised the data collection process.

### 2.10. Data Quality Assurance

Training was given for data collectors to familiarize them with data collection tools to understand the objective of the study, the flow of tools, and the content of tools and to ensure ethical issues during data collection. The questionnaire was pretested at Ello Erasho Health Center among 5% of the study subjects. Then, necessary adjustments and corrections were made by investigators to standardize and ensure its validity. The principal investigator and field supervisor were checking completeness and consistency of the questionnaire immediately after interview at the field level and before receiving the filled questionnaire.

### 2.11. Data Management and Analysis

The collected questionnaire was checked manually for its completeness and coded and entered into EpiData version 3.01 statistical package, then exported to SPSS version 23.0 for further analysis. Knowledge question responses were added up, and those who scored 50% correct answers and above were categorized under good knowledge. In attitude questions 301-305, “uncertain, disagree, and strongly disagree responses” were all recategorized to “disagree” and “agree and strongly agree” responses recategorized to “agree”; in attitude questions 306 and 307, “agree,” “strongly agree,” and “uncertain” responses were recategorized to “unfavorable attitude” and “disagree and strongly disagree” in these questions were recategorized to “favorable attitude,” then the mean was calculated.

Descriptive statistical calculations were done, and data was presented by table, pie chart, and graphs. Both bivariate and multivariate logistic regression analyses were used to determine the association between independent variables and dependent variables. Variables with *p* value less than 0.2 in bivariate analysis became a candidate for multivariate analysis. Multivariate analysis was done to adjust the effects of cofounders on the outcome variable. Odds ratios with their 95% confidence intervals were computed to identify the presence and strength of association, and statistical significance was declared if *p* < 0.05.

### 2.12. Ethical Consideration

This study was approved by the Research and Ethics Committee of Addis Ababa University, Department of Nursing and Midwifery. An official letter was taken from the department of nursing and midwifery of Addis Ababa University and submitted to the Wolaita Zone health office to obtain permission to carry out the study at the selected hospitals and health centers. Informed written consent was obtained from each study participant, before the interview. Any personal identification of the study participants was not recorded during data collection. Confidentiality of information was secured by keeping the questionnaires and data in a secured place.

## 3. Results

### 3.1. Sociodemographic Characteristics of Study Participants

A total of 269 obstetric caregivers participated in the study making response rate 97.8%, from 275 obstetric caregivers in the selected health facilities. About more than half (51.7%) of obstetric caregivers were males. The mean age of the respondents was 30.41 years (standard deviation (SD) = 5.029). Majority (59.5%) of them were working at the health center. Their profession (32.7%) was midwifery with a bachelor level of study as shown in Table 1.

### 3.2. Partograph Utilization

All 269(100%) of obstetric caregivers responded that they had used the partograph at least once to monitor labor. From these, 193 (71.7%) were routinely utilizing the partograph and 76 (28.3%) participants were not routinely utilizing the partograph.

### 3.3. Reasons for Not Utilizing Partograph

The main reasons of not utilizing the partograph routinely were unavailability of the partograph 45/76 (16.7%) and lack of training 34/76 (12.6%) followed by lack of supervision 29/76 (10.8%) on partograph utilization at their facilities.

**Figure 1:**
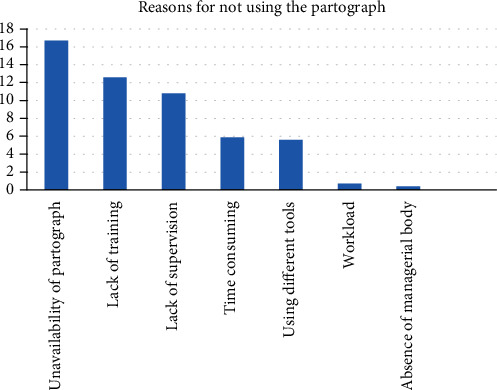
Reasons for not using the partograph by obstetric care providers of a public health institution in Wolaita Zone, SNNPR, Ethiopia, 2017.

In addition, 16 (5.9%) respondents reported difficulty to plot properly on the partograph because it is time consuming. On the other hand, 15(5.6%) of the participants were using different tools instead of the partograph as shown in Figure 1.

### 3.4. Knowledge and Attitude of Obstetric Caregivers towards Partograph Utilization

Concerning knowledge on utilization the partograph, more than half (61.7%) of the caregivers were knowledgeable (details of knowledge question responses are in Table 2). All of the participants learned about the partograph while at college or university, and 82.9% of participants received on-the-job training on the partograph. Agree and strongly agree responses were recategorized to “agree” and uncertain, disagree, and strongly disagree were recategorized to “disagree.” The majority (80.7%) of obstetric caregivers responded more than the mean and hence were categorized as having a favorable attitude towards the partograph as shown in Table 3.

### 3.5. Factors Associated with Partograph Utilization by Obstetric Caregivers

According to the result of bivariate logistic regression, age of respondents (>30 yrs. old) (COR = 11.30, 95%CI = 4.50, 28.38), respondent's service year (7 or more) (COR = 14.75, 95%95%CI = 5.97, 36.26), profession (midwife) (COR = 0.53, 95%CI = 0.15, 1.90), respondents being knowledgeable (COR = 5.53, 95%CI = 3.11, 9.83), with favorable attitude towards partograph utilization (COR = 4.82, 95%CI = 2.56 − 9.08), working in a hospital (COR = 1.85, 95%CI = 1.05, 3.26), and on-the-job training (COR = 0.06, 95%CI = 0.03, 0.13) became candidate for multivariate analysis.

Among variables entered into the multivariate analysis, service year, in-service training, knowledge, and attitude of obstetric caregivers towards partograph were significantly associated with partograph utilization. Those who had work experience greater than 7 years were about 5 times more likely to utilize the partograph than those who were less experienced (AOR = 4.93, 95%CI = 1.53, 15.88). Those obstetric caregivers who received in-service training on utilization of partograph for the management of mother in labor were about 84% more likely to utilize the partograph than those who have not gotten in-service training (AOR = 0.16, 95%CI = 0.06, 0.43).

In addition, those respondents who had good knowledge on the partograph and its utilization were about 3 times more likely to utilize the partograph than those who were less knowledgeable (AOR = 3.35, 95%CI = 1.61, 6.97). Furthermore, those who had a favorable attitude towards partograph utilization were about 3 times more likely to utilize the partograph than their counterparts (AOR = 2.99, 95% CI: 1.28-7.03) as shown in Table 4.

## 4. Discussion

This study tried to identify the level of partograph utilization and associated factors among obstetric caregivers in Wolaita Zone, Southern Ethiopia. Accordingly in this study, 193 (71.7%) utilized the partograph routinely and 76 (28.3%) of participants did not utilize the partograph routinely. Low work experience in the delivery unit during the year, not getting in-service training on management of labor, favorable attitude towards partograph utilization, and knowledge of obstetric caregivers on partograph utilization were factors significantly associated with partograph utilization.

However, this finding shows a higher rate of use than the studies conducted in the North Shoa Zone in the Amhara region (40.2%) (162), Jimma University Specialized Hospital (6.9%) [17], Amhara region (29%) [22], Niger Delta Region of Nigeria (32.6%-37.5%), Addis Ababa (57.3%) [23], and University of Calabar Teaching Hospital, Nigeria (13.6%). The differences between these findings might be due to trainings provided on the partograph, differences in the place of the study and participation of midwives in other studies, and different level of knowledge and attitudes of caregivers towards partograph utilization [11, 14, 15, 17, 24]. In addition, the other reason for difference from the study conducted in Nigeria could be the difference in the method of data collection procedure and large sample size [24].

In this study, the reasons for not using the partograph during labor were unavailability of the partograph, not getting in-service training, lack of supervision, absence of managerial policy, time consuming, and use of different tools. This finding is similar with the studies in North Shoa and University of Calabar Teaching Hospital, Calabar, Nigeria [17, 24].

Health caregiver's year of service was also associated with partograph utilization (AOR = 4.93, 95% CI: 1.53-15.88), i.e., those health caregivers whose service years were more than 7 years were 5 times more likely to use the partograph to monitor the progress of the labor. This finding is in line with a study which was done in North Shoa, Ethiopia [17] and is the same with a study done in the Niger Delta Region of Nigeria (*χ*^2^ = 4.818, df = 4, *p* < 0.05). This may be due to having exposure to different on-the-job experiences which helps them to develop empathy; long-time experience of partograph utilization makes them analyze the outcome of laboring mothers and makes them use the partograph efficiently.

Getting in-service training in the management of a pregnant mother in labor had a significant association with partograph utilization. Those obstetric caregivers who received in-service training in the management of a pregnant mother in labor were 84% more likely to utilize the partograph than those who have not received in-service training (AOR = 0.16, 95% CI: 0.06, 0.43). This finding contradicts with the study done in North Shoa, Northern Ethiopia (AOR = 2.86, 95% CI: 1.69, 4.86) and the study done in Amhara, Ethiopia [15, 16]. This might be due to the fact that obstetric caregivers who received in-service training on the management of a pregnant mother in labor and how to use the partograph during labor follow-up had better practice about the partograph than others that in turn improves their partograph utilization.

Knowledge on the partograph is also a factor associated with partograph utilization in this study. Obstetric caregivers who were knowledgeable about the partograph were about 3 times more likely to utilize the partograph than those who were not knowledgeable (AOR = 3.35, 95% CI: 1.61, 6.97). This is similar with the study done in North Shoa, Ethiopia (AOR = 3.79, 95% CI: 2.05, 7.03), and is also in line with the study done in the Niger Delta Region of Nigeria (*χ*^2^ = 32.298; df = 1; *p* < 0.05) [14] and also agrees with the study done in in the University of Calabar Teaching Hospital, Calabar, Nigeria (*χ*^2^ = 52.5, *p* = 0.00). This implies that having knowledge about the partograph is important to utilize the partograph during labor.

In addition, this study revealed attitude as another factor affecting partograph utilization. Those who had a favorable attitude towards partograph utilization were about 3 times more likely to utilize the partograph than those with a nonfavorable attitude (AOR = 2.99, 95% CI: 1.28-7.03). This finding goes in line with the study done in North Shoa, Northern Ethiopia (AOR = 2.35, 95% CI: 1.14, 4.87) and is also in line with the study done in the Amhara region, North Ethiopia [15, 17]. This could be due to the fact that, when people have good attitude towards something, they utilize it more frequently than those who are less interested, so that, in the current study, those obstetric caregivers with unfavorable attitude had less utilization of the partograph.

## 5. Conclusion and Recommendation

### 5.1. Conclusion

Partograph utilization among obstetric caregivers was common in the study area. Years of experience, getting in-service training on the management of labor, knowledge of the partograph, and a favorable attitude towards partograph utilization are independent determinants of partograph utilization. Reasons for not using the partograph during labor were unavailability of the partograph, inadequate on-the-job training access, lack of supervision, absence of managerial body support, and using other different tools.

### 5.2. Recommendation

#### 5.2.1. To Wolaita Zonal Health Office

- Zonal health office should avail an adequate amount of partographs for utilization
- Zonal health office should supervise obstetric caregivers on whether they utilize the partograph for every laboring mother or not
- In addition, training should be given to all obstetric caregivers
- Retaining of experienced obstetric caregivers should be considered by providing incentives, positions, and educational opportunities
- Monitoring and supervision of obstetric staff to ensure appropriate use of the partograph should be given the highest priority by every hospital administrator and primary health care unit leader

#### 5.2.2. To Obstetric Caregivers

- The partograph should be used for every woman in labor and be taken seriously by the caregivers, and it should be considered as a tool for diagnosing problems during the progress of labor. Care should be given with due attention by thinking of the oaths of the profession

## Figures and Tables

**Table 1:** Sociodemographic characteristics of obstetric caregivers in Wolaita Zone, SNNPR, Ethiopia, 2017 (*N* = 269).

Variable	Category	Frequency	Percent
Respondent's sex	Male	139	51.7
	Female	130	48.3

Marital status of respondents	Single	80	29.7
	Married	178	66.2
	Divorced	11	4.1

Respondent's age	20-24	28	10.4
	25-29	108	40.1
	30 or more	133	49.4

Health facility	Health center	160	59.5
	Hospital	109	40.5

Respondent's profession	Gynecologist	12	4.5
	General practitioner	9	3.3
	Health officer	36	13.4
	BSc nurse	43	16.0
	Diploma nurse	19	7.1
	BSc midwifery	88	32.7
	Diploma midwifery	62	23.0

Respondent's religion	Protestant	163	60.6
	Muslim	24	8.9
	Orthodox	71	26.4
	Catholic	11	4.1

Respondent's working experience (in years)	1-3	81	30.1
	4-6	117	43.5
	7 or more	71	26.4

On-the-job training on partograph	Yes	223	82.9
	No	46	17.1

**Table 2:** Knowledge of obstetric caregivers on partograph utilization in Wolaita zone, Southern Ethiopia, 2017 (*n* = 269).

Variable	Category	Frequency	
		Frequency	Percent
A salient feature of recording the whole process of labor	Yes^∗^	165	61.3%
	No	104	38.7%

Components of partograph: identification, assessment of fetal and maternal wellbeing, and assessment of labor progress	Yes^∗^	215	79.9%
	No	54	20.1%

Knowledge about the start time of plotting partograph: at 4 cm cervical dilation	Yes^∗^	215	79.9%
	No	54	20.1%

Frequency of plotting on partograph once active phase of labor started: once/30 minutes	Yes^∗^	182	67.7%
	No	87	32.3%

Cervical dilatation should be plotted on partograph every 4 hours	Yes^∗^	220	81.8%
	No	49	18.2%

Partograph is used to detect deviation from normal delivery that develop as labor as labor progress	Yes^∗^	202	75.1%
	No	67	24.9%

Types of client that needs partograph use: all mothers in active phase of labor	Yes^∗^	235	87.4%
	No	34	12.6%

^*^Correct response.

**Table 3:** Attitude of obstetric caregivers towards partograph utilization in Wolaita Zone, Southern Ethiopia, 2017 (*n* = 269).

Variable	Category	Frequency	Percent
To follow women in labor, using partograph is beneficial for the laboring women	Agree	237	88.1%
	Disagree	32	11.9%

The partograph is very favorable as it alerts skilled birth attendant of any deviation from normal	Agree	236	87.7%
	Disagree	33	12.3%

By using a partograph, health caregivers are able to identify problems and recognize complications early	Agree	235	87.4%
	Disagree	34	12.6%

Skilled birth attendant must use a partograph on every laboring mother	Agree	239	88.8%
	Disagree	30	11.2%

Using partograph enables health caregivers to perform essential basic interventions and make referrals to appropriate levels of care when necessary	Agree	240	89.2%
	Disagree	29	10.8%

Using partograph is not beneficial as the estimate it gives is exaggerated	Agree	24	8.9%
	Disagree	245	91.1%

Using partograph misleads management as the progress of labor and the partograph alert line are not aligned in most pregnant woman	Agree	23	8.5%
	Disagree	246	91.5%

**Table 4:** Bivariate and multivariate analyses of factors associated with partograph utilization among obstetric caregivers in public health facilities in Wolaita Zone, SNNPR, Southern Ethiopia, Feb. 2017 (*n* = 269).

Variables	Category	Partograph utilization		COR with 95% CI	AOR with 95% CI
		Yes	No		
Age of respondent	20-24	11	17	1	1
	25-30	65	43	2.37[0.99, 5.47]	1.41[0.48, 4.17]
	30 or more	117	16	11.30[4.50, 28.38]	2.47[0.69, 8.82]

Respondent's service year	1-3	31	50	1	1
	4-6	98	19	8.32[4.28, 16.18]	3.92[1.66, 9.23]
	7 or more	64	7	14.75[5.97, 36.26]	4.93[1.53, 15.88]^∗^

Respondent's profession	Medical doctors	18	3	1	1
	Health officer	22	14	0.26[0.07, 1.06]	0.269[0.04, 2.04]
	Nurse	39	23	0.28[0.08, 1.07]	0.28[0.04, 1.96]
	Midwifery	114	36	0.53[0.15, 1.90]	0.38[0.06, 2.32]

In-service training on management of labor	Yes	183	40	1	1
	No	10	36	0.06[0.03, 0.13]	0.16[0.06, 0.43]^∗^

Knowledge	Knowledgeable	141	25	5.53[3.11, 9.83]	3.35[1.61, 6.97]^∗^
	Not knowledgeable	52	51	1	1

Attitude	Favorable attitude	171	46	4.82[2.56, 9.08]	2.99[1.28, 7.03]^∗^
	Unfavorable attitude	23	30	1	1

Respondent's working place	Hospital	23	86	1.85[1.05, 3.26]	1.09[0.48, 2.46]
	Health center	53	107	1	1

^*^ *p* value < 0.05; 1 = reference point.

## Data Availability

All data will be available on reasonable request sent to Aseb Arba via email.

## Abbreviations

BEmONC: Basic emergency and obstetric care
EDHS: Ethiopian Demographic and Health Survey
FMOH: Federal Ministry of Health
HEWs: Health extension workers
IRB: Institutional review board
MDGs: Millennium Development Goals
MNH: Maternal and neonatal health
MMR: Maternal mortality rate
NGO: Nongovernment organization
OCGs: Obstetric caregivers
PHCU: Primary health care unit
PI: Principal investigator
SNNPR: Southern Nation Nationalities and Peoples Region
UNICEF: United Nation Children Fund
WHO: World Health Organization.

## Supplementary Materials

Supplementary Materials A pretested and structured questionnaire which was used to collect data is attached as a supplementary file.

## References

[1] World Health Organization (2004). *Beyond the numbers : reviewing maternal deaths and complications to make pregnancy safer*.

[2] UNFPA W *World Bank Group and the United Nations Population Division. Trends in maternal mortality. 1990 to 2015*.

[3] Central Statistical Agency Addis Ababa E (2016). *The DHS program ICF Rockville, Maryland, USA*.

[4] Abdella A. (2010). Maternal mortality trend in Ethiopia. *Ethiopian Journal of Health Development*.

[5] Opoku B. K., Nguah S. B. (2015). Utilization of the modified WHO partograph in assessing the progress of labour in a metropolitan area in Ghana. *Research Journal of Women’s Health*.

[6] Lavender T., Hart A., Smyth R. M. D. (2012). Effect of partogram use on outcomes for women in spontaneous labour at term. *Cochrane Database of Systematic Reviews*.

[7] Calvello E. J., Skog A. P., Tenner A. G., Wallis L. A. (2015). Applying the lessons of maternal mortality reduction to global emergency health. *Bulletin of the World Health Organization*.

[8] Sena Belina Kitila A. G., Molla A., Nemera G. (2014). Utilization of partograph during labour and birth outcomes at Jimma University. *Journal of Pregnancy and Child Health*.

[9] Weyesa J. B., Tadesse A. H., Eba T. Y., Minta M. K. (2015). Prevalence and risk factors associated with maternal mortality in Mizan-Aman hospital, Bench Maji, Southwest Ethiopia. *Women’s Health Care*.

[10] Mathai M. (2009). The partograph for the prevention of obstructed labor. *Clinical Obstetric Gynecology*.

[11] Fantu S., Segni H., Alemseged F. (2011). Incidence, causes and outcome of obstructed labor in Jimma University Specialized Hospital. *Ethiopian Journal of Health Sciences*.

[12] Navneet M. (2011). Partograph revisited. *International Journal of Clinical Cases and Investigations*.

[13] Qureshi Z. P., Sekadde-Kigondu C., Mutiso S. M. (2010). Rapid assessment of partograph utilisation in selected maternity units in Kenya. *East African Medical Journal*.

[14] Opiah M. M., Ofi A. B., Essien E. J., Monjok E. (2012). Knowledge and utilization of the partograph among midwives in the Niger Delta region of Nigeria. *African Journal of Reproductive Health*.

[15] Abebe F., Birhanu D., Awoke W., Ejigu T. (2013). Assessment of knowledge and utilization of the partograph among health professionals in Amhara region, Ethiopia. *Science Journal of Clinical Medicine*.

[16] Yisma E., Dessalegn B., Astatkie A., Fesseha N. (2013). Knowledge and utilization of partograph among obstetric care givers in public health institutions of Addis Ababa, Ethiopia. *BMC Pregnancy and Childbirth*.

[17] Wakgari N., Amano A., Berta M., Tessema G. A. (2015). Partograph utilization and associated factors among obstetric care providers in North Shoa Zone, Central Ethiopia: a cross sectional study. *African Health Sciences*.

[18] Orhue A. A. E., Osemwenkha A. P., Aziken M. E. (2012). Partograph as a tool for team work management of spontaneous labor. *Nigerian Journal of Clinical Practice*.

[19] Nyamtema A. S., Urassa D. P., Massawe S., Massawe A., Lindmark G., van Roosmalen J. (2008). Partogram use in the Dar Es Salaam perinatal care study. *International Journal of Gynecology & Obstetrics*.

[20] Ogwang S., Karyabakabo Z., Rutebemberwa E. (2009). Assessment of partograph use during labour in Rukumbural health sub district, Rukungiri district. *African Health Sciences*.

[21] Azandegbé N., Testa J., Makoutodé M. (2004). Assessment of partogram utilisation in Benin. *Santé*.

[22] Fahdhy M., Chongsuvivatwong V. (2005). Evaluation of World Health Organization partograph implementation by midwives for maternity home birth in Medan, Indonesia. *Midwifery*.

[23] Bosse G., Massawe S., Jahn A. (2002). The partograph in daily practice: it's quality that matters. *International Journal of Gynecology & Obstetrics*.

[24] Agan T., Akpan U., Okokon I. (2014). Assessment of the knowledge and utilization of the partograph among non-physician obstetric Care Givers in the University of Calabar Teaching Hospital, Calabar, Nigeria. *British Journal of Medicine and Medical Research*.

