# Supplementary material for: Partograph Utilization and Associated Factors among Obstetric Care Providers Working in Public Health Facilities of Wolaita Zone, 2017
Source: J Pregnancy. 2020 Jul 1;2020:3631808. doi: 10.1155/2020/3631808 (PMC7350161; doi:10.1155/2020/3631808)
Supplement: Supplementary Materials — A pretested and structured questionnaire which was used to collect data is attached as a supplementary file. [file 3631808.f1.docx]

**English questionnaire**

Good morning/afternoon, my name is_________ and I am one of the data collectors for the study being conducted by Addis Ababa University, College of Health Sciences, department of Nursing and Midwifery.

The objective of the study is to assess the magnitude of partograph use and factors that affect utilization of partograph among obstetric caregivers in public health institutions of Wolaita Zone, SNNPR, and Ethiopia. You are selected to be a participant of this study if you give me consent after you have understood the following information sheet:

There is no benefit, payment or possible risk associated with participating in this study except the time spent for responding to the questionnaire. All information given by you was kept confidential. Your participation is voluntary and you are not obligated to answer any question you do not willing to respond. If you feel any discomfort with the question, it is your right to drop it any time.

**Questionnaire for assessment of partograph utilization and associative factors in Wolaita public health institution SNNPR, Ethiopia 2017.**

| Part I. Socio-demographic characteristics of study participants in Wolaita Zone SNNPR 2017 (Select the most appropriate answer and tick one from the list below) | | | | | | | |
| --- | --- | --- | --- | --- | --- | --- | --- |
| NO | Questions | | | Response option | Code | | |
| 101 | How old are you? | | ___________years | | |  | |
| 102 | Sex | | 1. Female 2. Male | | |  | |
| 103 | What is your current marital status | | 1. Not married 2. Married 3. Divorced 4. Widowed 5. Other specify________ | | |  | |
| 104 | Religion | | 1. Protestant 2. Muslim 3. Orthodox 4. Catholic 5. Other specify________ | | |  | |
| 105 | What is your profession | | 1. Gynecologist 2. General practitioner 3. Health officer 4. Nurse (BSc) 5. Nurse diploma 6. Midwives BSc 7. Midwives diploma | | |  | |
| 106 | Currently working in | | 1. Hospital 2. Health center 3. Other, specify__________ | | |  | |
| 107 | Service year (in years) | | ______________years | | |  | |
| 108 | Did you receive any in service training in the management of a pregnant mother in labor? | | 1. Yes 2. No | | |  | |
| Part II. Knowledge related questions towards partograph utilization, in Wolaita Zone SNNPR 2017. (Please tick one for each statement below to indicate your response) | | | | | | | |
| 201 | Have you heard about partograph? | 1. Yes 2. No | | | | | |
| 202 | What is partograph? | 1. A tool to be used only in active phase of labor 2. A graphic methods of recording 1st stage of labor 3. A salient feature of recording the whole process of labor 4. Other specify_____________ | | | | |  |
| 203 | What are components of partograph? | 1. Assessment of Fetal, maternal wellbeing and assessment of labor progress 2. Others specify_________ | | | | |  |
| 204 | During attending women in labor, when do you start plotting on the partograph? | 1. When labor s diagnosed 2. At 4cm cervical dilatation 3. When complication is detected 4. At 3cm cervical dilatation 5. Other specify_________ | | | | |  |
| 205 | How often is it used once active phase of labor started? | 1. Once/30 Minutes 2. Once/Hour 3. Once/4 Hours 4. Once /6 Hours 5. Other specify______________ | | | | |  |
| 206 | Cervical dilation should be plotted on partograph every 4hrs. | 1. Yes 2. No | | | | |  |
| 207 | Partograph is designed to detect deviations from normal delivery that develop as labor progresses | 1. Yes 2. No | | | | |  |
| 207 | Type of client that needs partograph use | 1. Primigravida 2. Multiparus 3. All women in active phase of labour 4. On eclamptic patient | | | | |  |
| Part III Attitude related questions towards partograph utilization: Please, tick one box to indicate to what extents do you agree or disagree with the following statement | | | | | | | |
| 301 | To follow women in labor, using partograph is beneficial for the laboring women | 1. Strongly agree 2. Agree 3. Uncertain 4. Disagree 5. Strongly dis agree | | | | |  |
| 302 | The partograph is very favorable as it alert skilled birth attendant of any deviation from normal | 1. strongly agree 2. Agree 3. Uncertain 4. Disagree 5. Strongly dis agree | | | | |  |
| 303 | By using a partograph, health care providers are able to identify problems, recognize complications early. | 1. strongly agree 2. Agree 3. Uncertain 4. Disagree 5. Strongly dis agree | | | | |  |
| 304 | skilled birth attendant must use a partograph on every laboring mother | 1. Strongly agree 2. Agree 3. Uncertain 4. Disagree 5. strongly disagree | | | | |  |
| 306 | Using partograph enables health care providers perform essential basic interventions and make referrals to appropriate levels of care when necessary. | 1. Strongly agree 2. Agree 3. Uncertain 4. Disagree 5. strongly disagree | | | | |  |
| 307 | Using partograph is not beneficial as the estimate it gives is exaggerated. | 1. Strongly agree 2. Agree 3. Uncertain 4. Disagree 5. strongly disagree | | | | |  |
| 308 | Using partograph misleads management as the progress of labor and the partograph alert line are not aligned in most pregnant woman. | 1. Strongly agree 2. Agree 3. Uncertain 4. Disagree 5. strongly disagree | | | | |  |
| Part V Characteristics of Partograph utilization and Factors affecting utilization of the Partograph in labor monitoring. | | | | | | | |
| 401 | Have you been using partograph to monitor labor? | 1. Yes 2. No | | | | |  |
| 402 | If *Yes* to Q401, how often is it used? | 1. Routinely(for all laboring mother) 2. Occasionally 3. sometimes | | | | |  |
| 403 | If “occasionally” or “sometimes” to Q402, What would make difficult to use the partograph routinely when monitoring women in labor? (you can tick more than option) | 1. Non-availability of partograph 2. Using different monitoring tools 3. Lack of supervision 4. Time consuming to use 5. Workload 6. Lack of trained human power 7. Shortage of staff 8. Absence of managerial policy | | | | |  |
| 404 | Did you learn about partograph while you are in college/university? | 1. Yes 2. No | | | | |  |
